# Climate Moralities Offset: A Case of Formative Voluntary Carbon Markets

**DOI:** 10.1111/1468-4446.70000

**Published:** 2025-06-02

**Authors:** Tomi Lehtimäki, Kamilla Karhunmaa, Tapio Reinekoski, Arttu Manninen, Mikko J. Virtanen

**Affiliations:** ^1^ Institute for Criminology and Legal Policy University of Helsinki Helsinki Finland; ^2^ Department of Design Aalto University Espoo Finland; ^3^ Sociology, Department of Social Sciences University of Helsinki Helsinki Finland; ^4^ Faculty of Social Sciences Unit of Social Research Tampere University Tampere Finland; ^5^ Sociology Department of Social Science University of Helsinki Helsinki Finland

**Keywords:** carbon offset, climate change, morality, sociology of engagements, voluntary carbon markets

## Abstract

This article contributes to sociological scholarship on climate change by examining the development of the voluntary carbon offset market in Finland. While intended to address the collective challenge of climate change, voluntary carbon offsetting has faced criticism for commodifying emissions and shifting responsibility to specific actors. Enabled by voluntary carbon markets, emissions and climate impacts are attributed to companies and individuals, reflecting the idea that each entity possesses its ‘own’ emissions that they can choose to offset. However, this attribution does not happen on its own. The present study thus examines how the collective problem of acting on climate change is coordinated through particular moral engagements. We focus on the socio‐legal formatting of the voluntary carbon offset market in the context of Finland, a Nordic welfare state. We trace the trajectory of Compensate, a key Finnish offset provider whose activities sparked public controversy and led to criminal charges for violating the country's Money Collection Act as well as a legislative reform aimed at formalising voluntary offsets. The controversy centred on the nature of voluntary offsets and whether to consider them to be generally beneficial climate actions or self‐interested activities. Based on the theory of the sociology of engagements, our analysis shows how actors engage in moral and political coordination in order to foster and sustain engagements with climate change. More broadly, our case demonstrates that producing and facilitating engagement with climate change through a voluntary market is not merely a matter of implementing effective instruments and arrangements—leading ultimately to the individualisation of climate action—but a result of complex moral and socio‐legal formations. We conclude that the formatting of particularised climate engagements is a collectively produced process that necessitates an analysis of the shared moral coordination involved.

## 1 Introduction: From Individualisation to Particularised Emissions

Voluntary carbon markets (VCMs)^1^ have emerged as a new form of acting responsibly regarding the climate, making it possible for individuals to take care of their own emissions and for companies and organisations to reach their ‘carbon‐neutral’ targets. At the same time, VCMs have sparked debate in both sociology and related public discourse (Blok 2011; Dalsgaard 2016; Huff 2023; Karhunmaa 2025; Lovell et al. 2009; Paterson and Stripple 2010), with much of the recent discussion adopting a critical perspective. Voluntary offsetting has been accused of enabling the perpetuation of a business‐as‐usual approach whereby the global North can maintain a fossil‐fuelled way of life by making modest monetary payments for its emissions to be offset by others in the global South. The most intense condemnation of such voluntary offsets denounces them as a modern form of medieval indulgences (Dalsgaard 2022).

The technical arrangement of voluntary offsets is thus enmeshed with moral engagements, with particular actors viewed as buying themselves out of a moral and political issue (Franki 2022; Nerlich and Koteyko 2009; Paterson and Stripple 2012). Instead of treating climate change as a *general* problem that requires systemic solutions, voluntary offsetting directs attention to the *particular* emissions of different actors, whether individual persons or companies (Brill 2021; Lovell et al. 2009). A tension emerges between the collective goal of climate change mitigation and particularised engagements in which individual actors attempt to resolve a shared problem. For proponents, carbon offsetting is a legitimate way to do one's share to address a common issue, whereas critics see it as a form of greenwashing or indulgence that precludes an authentically involved moral engagement with climate change (Valiergue and Ehrenstein 2023).

In the present study, we target this moral dimension of VCMs and analyse how the collective problem of acting on climate change is coordinated through particular moral engagements. We draw on a case study of Compensate, a significant Finnish offset provider whose actions since its launch in 2018 ignited a public and legal controversy about the very essence of voluntary offsets. Central to the dispute was the question of whether voluntary offsets should be considered charitable contributions. Finnish regulators initially interpreted payments for voluntary offsets as contributions to the collective good rather than transactions or purchases. This resulted in criminal charges being pressed against Compensate for violating the Finnish Money Collection Act, which did not permit companies to collect money for the collective good. Further, it disrupted and temporarily suspended the functioning of Finland's VCM during the criminal process. What followed the charges was a public debate and legislative reform, which had to gradually disentangle, articulate and coordinate the relation between individual action and collective concerns emergent in voluntary carbon offsetting. The dispute therefore opened a broader debate about climate governance and the moral quality of voluntary offsetting, aiming to establish a shared understanding of what offsets were ‘really’ about.

To analyse the kinds of moralities that were enacted in the socio‐legal formatting of the VCM in Finland, we use the analytical framework of the sociology of engagements that builds on the theoretical program conceived by Laurent Thévenot (2007),  (2014),  (2015). Drawing on the sociology of engagements perspective allows us to focus on a process of *particularisation* as a key moral engagement related to the VCM. In this, emissions are allocated to *specific actors* through the socio‐legal formatting and coordination undertaken by the actors involved (cf. Affichard et al. 2023; Thévenot 2019). However, the particularisation of collective climate concerns through offsetting is not a straightforward process. We highlight three key aspects of the formatting of particularised engagements in the Finnish VCM: first, the construction of individual responsibility in the attempts by Compensate to mobilise actors into taking care of everyone's ‘own’ emissions; second, the legal categorisation of voluntary offsetting, which aimed to settle the relation between common and private benefits and define the nature of offsets; and third, the coordination of the regulatory environment geared toward supporting this form of action.

Our key contribution demonstrates how the controversy over carbon offsets compelled a reconfiguration of existing practices and an explicit social and legal rethinking of the meaning of collective and individual morality in efforts to mitigate climate change. As such, formatting and defining carbon offsetting as a distinct practice set apart from generally beneficial climate action did not operate as a linear process of implementing effective instruments and arrangements that would have led to the individualisation of climate action. More broadly, then, our analysis demonstrates that a central aspect in the coordination and formatting of the VCM is the construction of a morally sound justification for action as a reason to engage in offsetting.

In addition, our analytical work contributes to the development of the sociology of engagements. Although the framework specifically emphasises the formatting and construction of interests (Blokker and Brighenti 2011, 390; Thévenot 2020, 224–225), there have not been enough analyses focusing on these aspects. Therefore, we examine the ways in which interests are constructed instead of treating them as given attributes. We propose that future studies attend to legislative reforms in a similar way, as processes of formatting engagements and interests.

Before turning to our analysis of the development of the regulatory landscape of voluntary offsetting in Finland, in Section 2 we discuss VCMs and the various critiques directed at them through the sociology of engagements. Section 3 presents the analytical framework and our materials and methods. After presenting our three‐part analysis, we conclude our argument in the final section.

## 2 Voluntary Carbon Offsetting and Moral Engagements

Despite the growth of voluntary offsetting and associated hype, criticism of voluntary offsetting has been widespread. VCMs are contested based on their various technical configurations and the environmental credibility of carbon offset projects as well as due to the negative trade‐offs and consequences resulting from carrying out offset projects (Cavanagh and Benjaminsen 2014; Edstedt and Carton 2018; Erickson et al. 2014; Lovell and Liverman 2010). Broader concerns have also been raised about the unequal global distribution of climate responsibility and the moral implications of apportioning emissions (Böhm and Dabhi 2009; Lohmann 2006; Lovell et al. 2009). For these critics, carbon offsetting ‘permits societies in the global north to maintain luxurious lifestyles and to rely upon fossil fuel‐based unlimited growth rather than turning them toward the “true” alternatives’ (Dalsgaard 2016, 76).

Meanwhile, common strategies used to justify offsetting as a response to climate change have focused on the idea of a global climate that is indifferent to the origin of emission reductions. The feasibility of carbon offsetting builds on a specific yet universal measure: atmospheric carbon as a compound metric that forms a global whole divisible into smaller parts (Cooper 2015; Paterson and Stripple 2012). Through the common use of tonnes of carbon dioxide equivalent (tCO_2_e), which ‘makes things the same’ (MacKenzie 2009, 440), all climate actions can be made calculable and comparable for addressing climate change (Dalsgaard 2016). Perceiving all emissions as equal requires significant scientific, technical, institutional and political work, drawing on both environmental economics and atmospheric science (Callon 2009; Callon and Muniesa 2005; Shackley and Wynne 1995).

However, it is precisely this effective technical and market‐based allocation of emissions that has faced moral criticism—though of a different kind than that directed at the territorial shifting of burdens to the Global South. According to critics, solutions based on voluntary offsetting transfer the responsibility for addressing a fundamentally collective issue onto specific actors, including enterprises and individuals, who can act as both consumers of ‘carbon‐neutral’ products and services and directly purchase voluntary offsets (Bachram 2004; Franki 2022; Lohmann 2006). The technical calculations required to render carbon emissions commensurate and tradeable in VCMs construct an individualised climate subject that is responsible for managing its own portion of the emission burden (Blok 2011; Franki 2022; Paterson and Stripple 2010). Offsetting thus individualises climate change by offering monetised solutions that allow actors to avoid grappling with the full moral implications of their consumption and lifestyle choices.

The attempts to enable climate action and the ensuing criticism have led to VCMs being characterised both as a concerned and contested market (Ehrenstein and Valiergue 2021). Proponents regard their activities as a concerned engagement aimed at dealing with and even going beyond state‐led solutions to carbon emissions, while critics contest that view as greenwashing and sidelining the politics of a deeply value‐laden issue. Without taking either side in the debate, we argue that tensions between value registers in the enactment of VCMs warrant a thorough analysis, and the research tradition of the sociology of engagements has focused on precisely this kind of challenge.

Originally conceived by Laurent Thévenot (2007, 2014, 2015), the sociology of engagements presents a theory of how we compose a shared social world, coordinate disputes regarding it and strive to achieve commonality. The theory approaches commonality in a plural form, focusing on how actors actively engage with themselves and their environments and how they voice their concerns to others. Engagement refers to both a socio‐material connection with the world and a pledge that relieves uncertainties related to action and living together (Thévenot 2015, 2019). Thévenot and others have identified four *regimes* of engagement, each of which articulates a specific relationship to the world (or ‘reality’) and an associated form of good: public justification, focused on forms of common good; familiarity, focused on personal attachments; exploration, focused on excitement and the discovery of the new; and plan, focused on the functional use of the environment and promoting intentional, autonomous individuals (Hansen 2023). Each regime therefore outlines both a specific material engagement through which actors can grasp the world and a form of good that point to what is relevant (Thévenot 2002).

Furthermore, the regimes alone are not sufficient to construct commonality with others, and therefore the sociology of engagements also examines different *grammars* of commonality, each closely associated with a regime (Thévenot 2014). By relying on these grammars, actors can transform personal concerns into a common format and voice their concerns to others. In the grammar of plural orders of worth, concerns are formatted in relation to forms of common good, against which they can be evaluated and tested. In the liberal grammar, by contrast, concerns are formatted as choices and preferences among publicly available options (Hansen 2023, 12; Thévenot 2014, 16–17). Additionally, in the third grammar of personal affinities to common‐places, actors communicate issues with others through personal attachments, thus avoiding the public detachment and formalisation associated with the other two grammars (Thévenot 2014, 2020).

Engagement entails an ‘investment’ in a *specific form of acting together* (Thévenot 1984). Different ‘form‐giving operations’ are needed to construct a certain kind of engagement and to coordinate action (Thévenot 2019, 2). Here coordination ‘highlights the continuous and uncertain process of building categories and preferences’ in relation to an environment (Hansen 2023, 3). In following these form‐giving operations analytically, moral and political interests and qualities are seen as achieved through the formatting of engagements (Blokker and Brighenti 2011, 390; Hansen 2023, 5; Thévenot 2020, 224–225). Interests are therefore not treated as inherent, natural properties of actors driven exclusively by either self‐interest or the common good. Consequently, the coordination of the self is also ensured by a relation to the surrounding world that guarantees its continuity and the possibility of investing in acting together (Hansen 2023, 12; Thévenot 2014, 9, 2015, p. 92, 2019, p. 6).

Various studies have drawn on different elements of the overall theoretical program with a shared interest in the regimes and grammars through which we coordinate our relationships with ourselves and the environment, along with the tensions that arise in this coordination. Building on Boltanski and Thévenot's (2006) initial model of justifications, scholars have, for example, analysed various modes of ‘green’ justification and examined how the moral qualities of environmental engagements are tested (Foltyn et al. 2023; Lafaye and Thévenot 1993; Lehtimäki 2021; Lehtimäki and Virtanen 2024; Thévenot et al. 2000). Others have expanded the focus beyond what is collective and public to also include more personal forms of engagement and discussed different forms of coordination of the self (Blok 2015; Centemeri 2015, 2017; Luhtakallio 2019; Luhtakallio and Tavory 2018; Meriluoto 2023). Here, actors' practical activity is targeted as bridging the particular and the collective through the coordination of identities and ways of living together. For instance, in a study of climate activists and NGO workers, Luhtakallio and Tavory (2018) examine how actors align their identities by drawing on different regimes and thus transforming personal attainments into a form of collective coordination. The activists' identities intertwine with a form of collective engagement where ‘the mutual recognition of […] individual projects was the way to enact the common and the shared’ (p. 169).

Participation can also be coordinated by creating individualised arrangements that in turn affect actors' possibilities to voice their concerns (Cheyns 2014; Meilvang et al. 2018). In her study on the making of voluntary agricultural standards through ‘roundtables’, Cheyns (2014, p. 445) notes that such forms of engaging people gain their legitimacy by facilitating the ‘horizontal’ participation of different stakeholders but sacrifice claims referring to the common good. Consequently, the collective coordination and alignment of interests takes place through negotiations between different individualised formats. Engaging with such formats of collective effort therefore entails conflict and compromises, where some forms of acting together must be sacrificed for the sake of other kinds of ‘investments’ (e.g., Blok 2015, 129; Hansen 2023, 4–5).

Our analysis of the coordination of voluntary carbon offsetting builds on and contributes to this broad body of work on Thévenot's original program. We retain three key dimensions: a pluralistic view of action and forms of the good, the connection between the particular and the collective, and the tensions between different forms of engagement. By tracing the legal controversy and eventual legislative reform around the significant offset provider Compensate, we examine how regimes and grammars were interwoven to produce particularised moral engagements with offsetting and with climate change more broadly. We attend to the collective coordination of action and deploy the sociology of engagements to understand the transformation of issues into collectively recognised formats (Thévenot 2014, 2020).

## 3 Analytical Approach and Case

We set out to analyse the political and legal coordination of VCMs in the context of a Nordic welfare state. Our case study concerns a dispute about VCMs in Finland and the formatting of both voluntary offsets and the regulatory environment that builds on a variety of engagements (cf. Affichard et al. 2023; Diaz‐Bone et al. 2015). VCMs are by design shaped through voluntary commitments, treaties and legal arrangements that intertwine with techno‐economic frameworks (e.g., Blum 2020; Kreibich and Hermwille 2021; Lohmann 2006). Following Thévenot (2012), we argue that this formatting of VCMs is built on and thus carries a distinct emphasis on liberal individuality with a focus on individual rights, for instance, and the ‘individual's engagement in a voluntary plan’ that is ‘presupposed of the legal subject’ (Thévenot 2019, 6). In our case, this emphasis is reinforced by the connection between markets and the law (Bessy 2015; Knoll 2015).

We therefore focus on the legal dispute and reform and on the public discussion surrounding the controversy as processes of formatting in which actors rely on different engagements to coordinate actions (Diaz‐Bone et al. 2015). The sociology of engagements approach allows us to examine the socio‐legal and moral coordination of voluntary offsetting in a broader scope than mere blanket individualisation by also bringing into view forms of commonality operating ‘below’ the regime of justification (Cheyns 2014; Meilvang et al. 2018; Thévenot 2020). The framework allows for analysing how actors themselves engage in critique (Hansen 2016, 2023). Following this approach, we examine how actors engage in the coordination and formatting of the VCM.

We position our study in the context of Finland, an industrialised Nordic welfare state known for its corporatist and consensual policies (Arter 2013). Finland's distinctive characteristics make it an intriguing setting for investigating voluntary carbon offsetting. In Finland, contributions to the common good have historically been facilitated through progressive taxation and the establishment and consolidation of a robust welfare state. Additionally, Finnish climate policy in the 2010s has been guided by the explicit goal of achieving carbon neutrality, with a focus on bolstering international competitiveness, economic growth and innovation in Finnish companies (Karhunmaa 2021).

In this context, voluntary carbon offsetting has emerged relatively recently. During the early years of the millennium, only a handful of companies, such as Nordic Offset (established in 2008), offered voluntary carbon offsetting sourced from international offset projects to consumers and businesses. The public discussion surrounding offsetting gained traction in the late 2010s with the launch of the Compensate Foundation in 2018 and its corporate analogue a year later. Compensate immediately drew public attention because it was led by Antero Vartia, a former Green Party Member of Parliament and prominent public figure, and soon enough its activities sparked a controversy and a legal dispute regarding the nature of voluntary offsets. The question of the legality of offsets revolved around whether they were a form of charity that would have required a permit to collect money for a common cause, a permit that for‐profit firms could not obtain. The controversy and ensuing legislative reform created a situation that brought together a variety of actors who engaged in defining the moral, regulatory and political character of voluntary offsets to settle the dispute. We focus particularly on Compensate because—while it was by no means the only actor involved—its actions were widely publicised and led to significant legislative changes, as we show below.

To build a comprehensive understanding of the socio‐legal development of Finland's VCM, we draw on a dataset that has followed the evolution of the VCM in Finland since 2018 (Table 1). For this article, we have focused specifically on the material that deals with Compensate, the surrounding media debates, the associated legal disputes, and the regulatory reforms that followed.

**TABLE 1:** Materials used in the analysis.

Sources	Time frame/period	Approach
News articles^a^ (media discussion?)	2018–2023	Public discussion, defining individual responsibilities and interests
Legislative reform	2019–2021	Legal definition, classification and categorisation of voluntary offsets
Compensate's materials	2019–2022	Public communication, defining the meaning of voluntary offsets
Reports and seminars	2020–2023	Regulation of voluntary offsetting, regulatory environment

^a^ We collected materials published in wide‐circulation media related to Compensate and voluntary offsetting in general, which enabled us to analyse the public framing of voluntary offsetting and the legal controversies surrounding Compensate's activities. The four news outlets were chosen to represent national broadcasting (YLE), the most widely distributed newspaper (Helsingin Sanomat), a newspaper centred on rural issues (Maaseudun Tulevaisuus), and a business‐focused newspaper (Kauppalehti).

To examine in detail how voluntary offsets were defined and categorised in the materials—and to uncover the forms of engagement underlying those definitions and categorisations—the entire research team conducted a collaborative interpretative analysis. Initially, the first and third authors performed a preliminary content analysis of the data, compiling quotations related to Compensate's trajectory within the broader socio‐legal development of the VCM. This phase was followed by a theoretical interpretation by the first author, who then draughted the initial versions of the article's analytical sections. These draughts were subsequently further analysed by the entire research team in the light of theoretical insights and prior research. Finally, drawing on this collective interpretation, the first author developed a three‐part analytical scheme and integrated its components to interpret the findings.

Below, we present our analysis in three subsections that trace the chronological development of the case in light of the conceptual framework of the sociology of engagements. In so doing, we seek to understand the tension between a concern for the collective good of climate change mitigation and particularised moral engagements with offsetting.

## 4 Analysis

### 4.1 Cleaning Up Your Own Mess: Constructing Individual Responsibilities in Compensate's Communications

The Finnish offset provider Compensate has consistently referred in its communications to the necessity of taking individual responsibility in the face of a collective crisis. One central metaphor referred to in several online seminars and news articles was spilling a glass of milk and understanding that no one else will come to clean up the mess—we all need to clean it up ourselves. In an online seminar on 8 April 2021, Compensate founder Vartia regarded it as ‘astonishing that we still expect someone else to clean it up’, referring to the idea of expecting others to act on our behalf. Another metaphor also related to cleaning up a mess was littering in a park. This image emphasises the need to take *additional* action instead of just avoiding harmful activity: ‘If I throw trash in the park every day, I can't solve it by just throwing trash every other day. We need to have proper tools to take care of it, to clean it up’ (Seminar, 8 April 2021). Voluntary offsetting was contrasted to reducing emissions (or littering less frequently) and claimed to enable better tools for cleaning up the already caused mess.

Such metaphors cast voluntary offsetting as additional and purposeful climate action that comes on top of the need to reduce emissions. The aim is to articulate personal responsibility in a concrete way, thus making the crisis real for every individual. In a radio interview the year after Compensate was established (Kenttämaa and Vettanen 2019), Vartia spoke of the importance of taking individual responsibility. When asked about the price of offsets, Vartia noted that it was indeed important to make environmental damage both visible and calculable to consumers. But this was only part of the picture:

What I think is even more important here is that people would learn to understand that we are causing damage and that this damage has a price. And if I won’t pay when I consume something that produces damage, I’m assuming that someone else will, and is that right then? And if no one pays, then climate change will just keep on going and will cause tremendous damage and will certainly cost us dearly.

(Kenttämaa and Vettanen 2019)

Taking individual responsibility contrasts with what Vartia termed the ‘outsourcing of responsibility’, which distinguishes between individualised responsibility and various collective solutions:

We’re mentally *outsourcing the responsibility*. Each of us causes damage, yet we fail to recognise the fact and assign the blame to politicians. Politicians blame everyone outside their borders. The circle of avoiding responsibility is endless. And as long as it remains so, we will fail to effectively mitigate climate change.

(Compensate 2021b, 6; emphasis added)

In this moral framing, individual responsibility is real, in contrast to abstract and intangible engagements with the common good. What is considered real is not achieved through generalisation or abstraction to the level of the global atmosphere or collective decision‐making but through the proposition that people will understand very concrete examples of the harm they do and the costs they incur. In this view, only when people are obliged to recognise their own actions and accept responsibility will there be meaningful action. The collective solution thus consists of an aggregation of individual responsibilities. This liberal grammar in Compensate's communication is emphasised in how it created a contrast with lagging state‐led negotiations. The company does not dispute the overall need for collective or binding agreements or politics, but rather formats offsetting as a complementary alternative that helps individuals and companies carry their share of the burden and do their part. In this way, voluntary offsetting is kept apart from and treated as additional to collective or political solutions.

Furthermore, Compensate presents voluntary offsetting as a supplementary option by referring to the so‐called mitigation hierarchy for acting on climate change or deploying offsetting only as a last step after emission reductions. According to that hierarchy, actors should always first try to avoid producing emissions; second, they should aim to reduce emissions; and third, and only as a last resort, should they offset emissions. The mitigation hierarchy therefore presents a kind of order of worth or tool for valuation that frames offsetting as the *least* valuable option, to be used only as one among other complementary options (Lovell et al. 2009). Framed in this way, voluntary offsetting therefore presents a different kind of *critique* than what is commonly analysed from the perspective of the sociology of engagements (Hansen 2023, 7; Thévenot 2011, 54). In what could be termed a ‘liberal critique’, the alternative to collective negotiations is offered in a double move where the critique both points out that collective negotiations are problematic and at the same time affirms them as necessary. This critique does not fundamentally challenge other alternatives, as would be the case in the regime of public justification, but seeks to legitimise offsetting as a viable option with which actors can choose to engage (Cheyns 2014, 445).

In various publicity materials and seminars, Compensate took other VCM actors to task for failing to abide by the mitigation hierarchy and being driven by the pursuit of profits from selling offsets. Compensate repeatedly referred to its rigorous evaluation criteria and impact scores through which offset projects were put to the test. Questions about the integrity of VCMs were defined as ‘market flaws’ that primarily concerned issues in the design of offset projects and the market. This critique, situated in the grammar of plural orders of worth, can be understood as a green (or green‐market) evaluation that aimed to distinguish Compensate's projects as more environmentally sound than those of its competitors (Compensate 2023). Through both these public and liberal critiques, Compensate sought to differentiate itself from others by positioning itself morally as providing a necessary option, even if one that was suboptimal on the whole.

The liberal grammar emphasises individual responsibilities and their reciprocity and that issues be formatted as choices, options and interests (Thévenot 2011, 2014). Voluntary offsetting provides the opportunity to form an engagement that can bypass the complex negotiations required to reach agreement with others. This necessitates the formatting of options from which actors can choose. That is, before actors can make any choices, those options must be available to those actors. Furthermore, they need to make sense. If given the option to act and clean up, all actors should take care of *their own* emissions, as the title of one of Vartia's interviews suggests (Kenttämaa and Vettanen 6 May 2019). Taking part is framed as a normalised—even self‐evident—action, as illustrated by Vartia's rhetorical question in a social media video: ‘Why wouldn't we take responsibility?’ (Vartia 2019). At the same time, it hides the work done to construct the self‐evident character of such choices. Therefore, in addition to technical arrangements (e.g., MacKenzie 2009), equivalencies in voluntary offsetting are based on constructed individual responsibilities in which each actor has not merely the possibility but a moral obligation to take action. The construction of choices according to liberal grammar shapes actors into the format of responsible, calculating individuals who have internalised the responsibility that comes with the ability to choose.

However, the legal nature of the relation between the responsibility of the individual and the benefit for a wider collective became a subject of controversy soon after Compensate started operating in Finland. This public dispute revolved around the question of the motives, interestedness and potential rewards gained by actors engaged in the VCM. We turn next to how the relation between individuals and wider collectives was settled legally.

### 4.2 Defining Voluntary Offsetting: Legal Categorisation of the VCM in the Legislative Reform

On 31 December 2019, the newspaper *Helsingin Sanomat* reported that the National Police Board had filed a request for an investigation into Compensate's activities, as there were suspicions of violations of the Money Collection Act (Pikkarainen and Hartikainen 2019). In the request, offsetting was interpreted as a charitable activity that consequently required a fundraising permit. However, since it was not possible for a company to obtain a fundraising permit in Finland under existing legislation, Compensate's activity should be considered illegal. The National Police Board officials first interpreted offsetting services as charity because consumers purchasing offsets were not viewed as taking part in a regular market transaction in which they would gain ownership of something. Instead, protecting the global climate contributes to the common good, as emission reductions are not limited to a particular place, and consumers only receive a promise that their money will be used to reduce emissions (it does not grant, say, ownership of the trees planted to reduce emissions).

However, numerous experts disputed the National Police Board's interpretation as a ‘complete misunderstanding’ of the nature of VCMs. In its statement during the hearing process, Compensate (2021a) described the act as being completely outdated, ‘absurd’ and facilitating ‘arbitrary’ and ‘high‐handed’ decision‐making by officials. The difference between charity and purchasing carbon offsets was treated as arising from the existence of carbon credits that circulate in markets and thus have a market value. In the ensuing media debate, VCMs were explicitly framed by economists as enabling individual responsibility. For example, Markku Ollikainen, a professor of environmental economics who was then chairman of the Finnish Climate Panel, viewed the National Police Board's interpretation of voluntary offsets as charity as completely misguided:

The interpretation that people would be buying just good spirits here is completely false. It’s obvious that the National Police Board does not understand the nature of global emissions. If I buy a goat for someone as a gift, I’m not compensating for a harm I’ve caused. In carbon offsetting I am compensating for the harm I’ve caused others.

(Teittinen and Helsingin Sanomat 2020)

Here, voluntary offsets are transactions between individual actors who are not merely seeking these ‘good spirits’ but to compensate for harms caused. While the harm is implicitly described as inflicted on others as a collective, the actors do not engage in offsetting in order to contribute to a collective good. Instead, one offsets one's own emissions and thus takes individual responsibility.

The criminal case against Compensate and the public outcry it spurred led the Minister of the Interior, Maria Ohisalo (Green Party), to initiate a process to reform the law in 2020. In a media interview, she stated that if current legislation did not allow for carbon offsetting, the Money Collection Act should be changed (Maukonen 2020). In other words, the law needed to be modified to facilitate the consolidation of the VCM, not the other way around. As with the discussion in the previous subsection, the actors here do not aim to rise to the level of general principles and engage in evaluating the situation. The continuation of the planned action is considered primary and is to guide how the regulatory environment must be formatted. The legislative reform therefore represents the work needed to format the regulatory environment that is connected to the construction of interests.

The main aim of the reform was to clarify and settle concerns regarding the nature of voluntary carbon offsetting. Ambiguities related to the legal nature of VCMs had led to concerns about blurring the line between charitable donations and market transactions. This obscuring was amplified by the perception that offsetting was gradually becoming viewed as a term associated with all types of ‘activities generally benefitting the climate’ (Finnish Government 2021, 19). Several stakeholders interviewed by the Ministry of the Interior for the legislative reform regarded it as essential to differentiate voluntary offsetting as a specific transaction from other generally climate‐benefitting actions. In effect, the reform set these two forms of climate action apart, with one focused on the general public good and voluntary offsets defined as a specific arrangement. In the reform proposal, climate‐related fundraising was defined as charitable, something directed to an unspecified public that was based on citizens' desire to help in some way. This was differentiated from voluntary carbon offsetting as an arrangement between specific parties engaged in transactions (Finnish Government 2021, 8). The reform thus placed VCMs outside the scope of the Money Collection Act. Overall, it operated as a process of ‘building categories and preferences’ (Hansen 2023, 3) that aimed to define voluntary offsetting through individual interests. The formatting of interests according to the liberal grammar was connected to the structuring of the regulatory environment that supported voluntary offsetting.

Although the initial controversy concerned the moral character of voluntary offsetting—that is, whether it was a market transaction or a form of charity—the eventual conclusion during the legislative reform was that the interests behind offsetting could not be used to define offsetting in general. This was because NGOs and businesses wanted the option to engage in offsetting as both producers and purchasers of offsets. Therefore, while establishing the conceptual demarcation between the two spheres of activity—generally beneficial activity in civil society and commercial activity—the definition of voluntary offsets was based on the measurability of offsets and the assumption that they would somehow be calculable or at least realistically estimated. Instead of an activity that was generally beneficial to the climate, offsetting was about particular events when specific, calculable emissions were offset. The definition of this ‘offset event’ was the legal‐technical way to describe and demarcate voluntary offsetting as a specific form of activity. There was also consensus among stakeholders that voluntary offsetting should be made possible for all kinds of actors, whether they were nonprofit associations, businesses or individual people. Together with other changes, the definition of the offset event therefore formatted voluntary offsets as a publicly available option for all actors to choose if they wanted, placing them all on the same level (cf. Cheyns 2014; Thévenot 2015).

The new definition paid no attention to the quality and trustworthiness of offsets, which were demarcated outside the scope of the new act. Once the reformed Money Collection Act was passed in 2021, the criminal charge against Compensate was dropped. Since the new law took effect, public officials have engaged in attempts to coordinate the developing market. These regulatory attempts demonstrate another respect in which the voluntary nature of VCMs is maintained and to which we turn next.

### 4.3 Regulating Voluntary Offsetting ‘From a Distance’: Information Steering and Good Practices

The regulatory measures developed in the wake of the legislative reform aimed to tackle uncertainties and increase trust in offsetting measures. During the Money Collection Act reform, there was a consensus among various actors in the field that additional regulation was needed to clarify practices related to voluntary offsetting and to promote growth in the market by creating stability. While that coordination has largely been led by the responsible ministries, the Ministry of the Environment (MoE) and the Ministry of Agriculture and Forestry (MoAF), it has also involved actors like research institutes, financial institutions, offset companies and consultants. This is typical of the development of markets and the crafting of public policies through the involvement of ‘cadres of certification agencies, consultants and experts’ from whom policy gains part of its legitimacy and objectivity (Meilvang et al. 2018, 17; see also Knoll 2015).

In a report commissioned by the MoE, the making of offset claims is coordinated through the notions of accuracy and measurability (e.g., Laine et al. 2021, 60), which operate according to the principles of the industrial order of worth. The identified concerns were thus focused on whether voluntary offset markets would be the most efficient way to reduce emissions. The focus was primarily on the consumer perspective, with an aim to provide people with enough information to make informed decisions about the offsets available. National regulation coordinated by different ministries has quite understandably been focused on the Finland‐wide collective, but what is noteworthy here is that this coordination nevertheless aimed to minimise additional regulations, focusing more on securing the freedom of different actors to engage in offsetting.

The various regulatory options considered were command‐and‐control efforts based on new laws, economic incentives, industry self‐regulation and information steering (Laine et al. 2021). Overall, the report took the stance that minimising regulation was the most beneficial solution for society in general (Laine et al. 2021, 65). Thereby, all legislative options and economic incentives were regarded as unsuitable for VCMs; instead, guides and other forms of information steering, along with facilitating optional or voluntary ‘green deals’ and ‘pledges’, were considered desirable. Assessing possible regulation options thus operated from the perspective of voluntary actions in which actors aim toward a set goal: ‘Purchasing voluntary offsets is about voluntary environmental protection and not about fulfilling mandatory responsibilities’ (Laine et al. 2021, 87).

One example of this tension in the modes of coordination is visible in the discussion of a proposed national climate register:

In order for the [suggested] register to fulfil its aims, the requirements for admission should be high enough in order for the register to facilitate the identification of trustworthy and responsible actors. On the other hand, if the requirements are too high, the increased costs will decrease the willingness of Finnish actors to register in it.

(Laine et al. 2021, 83)

This presented an issue of quality versus quantity, with the report eventually deciding that more actors engaged in offsetting is better than facilitating a smaller number of high‐quality projects (cf. Valiergue and Ehrenstein 2023).

Another form of information steering was to establish ‘good practices’ for the VCM to coordinate and support its growth. These good practices were connected to the conceptual formatting of voluntary offsetting, with officials defining a set of concepts in Finnish related to the practice. The guidebook was written by consultants and published by the MoE (Laine et al. 2023) and set out to define terms in Finnish for the activities and units in VCMs. For example, it described voluntary climate action generally as those activities that support climate mitigation outside a company's own value chain. In this effort, officials aimed to coordinate the moral elements related to VCMs, as the terms limit the kinds of claims or promises companies should make and the types of actions that should be carried out in the market. In aiming to clarify and coordinate the claims that actors can make, the guidebook operates as a sort of etiquette manual that defines good conduct and appropriate claims made in the VCM. In addition to the publications, the MoAF financed the development of an online information platform, and both the MoAF and MoE have organised several seminars and other events to disseminate the newly developed terminology.

Different forms of *information steering* are thus part of how the voluntary nature of this engagement is maintained. The market is kept at bay as a voluntary and additional supplement to governmental climate action, and regulation is built on the hope that both consumers and providers will take that information into account. Therefore, regulation operates as a form of ‘governance from a distance’, with distance understood as including a moral aspect because it is constructed around the autonomy of the voluntary actors that it simultaneously maintains (cf. Thévenot 2020). As actors cannot be forced to engage in offsetting—they must take part ‘voluntarily’—they can only be offered information and best practices, which they will ideally take into account, and which will eventually lead to the development of a market that can truly self‐regulate. This supports the engagement in planned action, which does not aim to mobilise actors to undertake common efforts but instead to provide the means for the continuation of their individual planned projects (Meilvang et al. 2018, 27; Thévenot 2007, 417, 2014, p. 13). Such a regulatory arrangement would, in this view, lower the threshold for providing offsets and yield a larger volume of them, ostensibly leading to greater emission reductions, as opposed to restricting the range of products to only a few providers of supreme quality.

## 5 Conclusion

Climate change is commonly considered a collective problem par excellence, necessitating a critical examination of our current ways of living and the actions required to change them (Franki 2022; Kreibich and Hermwille 2021). However, collective problems and actions are also coordinated through engagements where the collective whole is apportioned into particularised responsibilities. Technical procedures and devices like carbon footprint calculators provide means to attribute emissions to individuals, but they alone are insufficient and are combined with moral engagements that aim to shape people's interests and guide their choices. Furthermore, this is not done by market actors alone but operates through a collective coordination of action where, for example, companies are joined by a variety of actors like legislators and researchers (cf. Asdal 2014; Knoll 2015).

In analysing the moral and practical coordination of the Finnish VCM, our examination brought out three dimensions regarding how it was shaped into a particularised engagement. First, it was necessary that all societal actors be convinced to take care of what was defined as their share of the climate burden. This emphasises an approach to taking responsibility for climate action as a divisible part of a total sum: the collective responsibility of all the spilt milk requires each and every one to clean it up. The collective coordination of climate action operates through the reciprocity of individual or particular responsibilities that form the bonds between actors (Meilvang et al. 2018; Thévenot 2011). These bonds make comparisons and negotiations between individuals possible but at the same time keep individuals and the actions they pursue separate (cf. Dalsgaard 2016; MacKenzie 2009). While this might render emissions more concrete in certain contexts, it overlooks the fact that not all emissions are attributable to particular actors in a straightforward manner. This approach relies heavily on the techniques and practices of calculating and assigning emissions and does not neatly account for individuals' share of collectively produced emissions.

Second, the dispute concerning the character of Compensate's activities and the ensuing legal reform shifted the focus on the level of defining the nature of voluntary offsets and advancing an emerging market. The legal categorisation was initially focused on formatting the interests associated with voluntary offsetting. This categorisation established a distinction between generally beneficial climate action and voluntary offsetting, with the latter associated with an offset event in which a specific, measurable amount of emissions is counterbalanced. The legislative reform facilitated the possibility for all actors to offset their own emissions, thus ostensibly taking care of their climate impacts. This shaped voluntary offsets as a publicly available option for all actors. The problems related to the definition and legal categorisation of voluntary offsetting bring forth the ambiguous status of the VCM as a moralised market (Asdal et al. 2023; Fourcade and Healy 2007; Valiergue 2019), as it is simultaneously situated in the context of generally beneficial climate action (i.e., the collective, the common good) and particularised actions in specific private settings. The form of engagement we have analysed in this paper builds on this tension, where actors construct commonality by engaging in it as discrete individuals, taking care of ‘their own’ emissions (cf. Cheyns 2014). Simultaneously, the collective is kept at bay and particularised engagements preclude the construction of a common ‘we’.

Third, voluntary offsetting has been coordinated through the soft regulation of information steering to solidify the central terms of the market. While the previous two themes focused on how particular actors are attributed with both responsibilities and interests, this approach differs in that it leaves actors vague and even undetermined. Coordinating a voluntary market requires that actors indeed have legitimate free choice and are independent, which limits the possibilities of any further regulatory interventions in the market (Meilvang et al. 2018). This approach defines actions as voluntary, and action is coordinated through the formatting of the regulatory environment. The engagement connects this sense of reality—that is, making emissions and harms visible and actual through concrete calculations and metaphors—with a specific moral character of human beings. Relying on abstract negotiations on a general level is defined as ‘outsourcing responsibility’, whereas individual interests and responsibilities are presented as what people care about and what will spur action (Thévenot 2015, 92). By the same token, charitable actions are just ‘buying good spirits’, whereas voluntary offsetting is connected to actual harms and the relations between actors.

Within the liberal grammar, voluntary offsetting becomes contrasted with established conventions of climate change, such as collective decision‐making (Ylä‐Anttila et al. 2018). The resulting format of VCMs thus came to be defined based on its distinctiveness from other forms of climate action (Lehtimäki and Virtanen 2024). This diverges somewhat from how Thévenot (Thévenot 2011, 53–54) approaches relations between regimes. What we characterise as the liberal critique does indeed undermine the form of good related to other regimes—in this case the common good of collective climate negotiations—but only to a certain extent. This is because the liberal critique defines all alternatives as options and choices, thus avoiding confrontations that could lead to generalisation and a move to the regime of justification (cf. Blokker 2011; Hansen 2016, 2023). We suggest that this strategy may be one reason why the public discussion and ensuing regulatory measures did not raise the question of whether voluntary offsetting is in fact a desirable—let alone effective—way to mitigate climate change. The absence of explicit critique on the level of generality allowed the socio‐legal formatting of voluntary offsetting to avoid questions about the principles on which meaningful climate action should be based. Locking the focus on functional, planned actions thus facilitated a technically oriented and consensual reform of the Money Collection Act.

Yet the formatting of the Finnish VCM demonstrates how this was not an unproblematic process of an inevitably advancing individualisation. One might assume that the possibility of voluntarily offsetting one's emissions, supported by personal carbon footprint calculators, already implies that everyone has a personal responsibility to engage in mitigating actions. However, our case brings out the moral and socio‐legal coordination that was required to produce and facilitate this form of engagement (cf. Paterson and Stripple 2012). In addition, our analysis demonstrates the variety of actors and arenas—beyond the market and its core actors—that are involved in formatting this engagement with climate change in VCMs (Asdal 2014).

Here, the sociology of engagements proves useful in integrating the various aspects connected to the particularisation of emissions while also demonstrating their heterogeneity. Furthermore, it is useful in identifying the liberal mode of coordination, operating ‘between’ the level of publicity and familiar engagements (Blok 2015; Foltyn et al. 2023; Thévenot et al. 2000). We have shown how technical arrangements and particularised responsibilities were advanced through regulation and law (cf. Affichard et al. 2023; Diaz‐Bone et al. 2015). Focusing on the collective making of particular engagements is necessary, we argue, for understanding the processes by which VCMs are differentiated from other forms of climate action. This study thus contributes to understanding how environmental issues are individualised by widening the analytical perspective to processes of particularisation (e.g., Dalsgaard 2022; Franki 2022; Paterson and Stripple 2010). Analysing how carbon offsetting is formatted reveals how seemingly self‐evident ideas—like the harms of individual actions—are tied to general moral and political regimes and grammars for coordinating climate action.

## Data Availability

Data sharing is not applicable to this article as no new data were created or analysed in this study.

## Endnotes

^1^
VCMs are markets where individuals, companies, organisations and states can purchase carbon credits, or carbon offsets, to compensate for their climate impacts. The markets function as baseline‐and‐credit schemes, where the credits are produced in projects that compare emission reductions to counterfactual baseline scenarios (Ehrenstein and Muniesa 2013; Lovell and Liverman 2010). Most voluntary offset projects are located in the Global South, where emission reductions have been argued to be cheaper to undertake. The carbon offsets produced are bought by entities interested in making claims related to their climate impacts, such as terming themselves or their products carbon‐neutral, net‐zero, climate‐positive and so forth. VCMs are regulated through a variety of local, national and international frameworks and intersect with compliance carbon markets, such as the European Union's Emissions Trading Scheme.
